# Accumulation, Biotransformation, Histopathology and Paralysis in the Pacific Calico Scallop *Argopecten ventricosus* by the Paralyzing Toxins of the Dinoflagellate *Gymnodinium catenatum*

**DOI:** 10.3390/md10051044

**Published:** 2012-05-09

**Authors:** Amada Y. Escobedo-Lozano, Norma Estrada, Felipe Ascencio, Gerardo Contreras, Rosalba Alonso-Rodriguez

**Affiliations:** 1 Laboratorio de Biotoxinas Marinas, Instituto de Ciencias del Mary Limnologia, Universidad Nacional Autonoma de Mexico, Apdo. Postal 811, Mazatlan, Sinaloa 82040, Mexico; Email: amadayerene@yahoo.com.mx; 2 Departamento de Ingenieria Quimica-Bioquimica, Instituto Tecnologico de Mazatlan, Calle Corsario 1 No. 203, Col. Urias, Mazatlan, Sinaloa 82070, Mexico; 3 Departamento de Fisiologia, Biofisica y Neurociencias, Centro de Investigacion y de Estudios Avanzados del IPN, Av. Instituto Politecnico Nacional 2508, Mexico City, D.F. 07300, Mexico; Email: normaestrada9@gmail.com (N.E.); rcontrer@fisio.cinvestav.mx (G.C.); 4 Departamento de Patologia Marina, Centro de Investigaciones Biologicas del Noroeste, Mar Bermejo 195, Col. Playa Palo de Santa Rita, La Paz, B.C.S. 23096, Mexico; Email: ascencio@cibnor.mx

**Keywords:** *Argopecten ventricosus*, *Gymnodinium catenatum*, histopathology, paralyzing shellfish poison, paralysis

## Abstract

The dinoflagellate *Gymnodinium catenatum* produces paralyzing shellfish poisons that are consumed and accumulated by bivalves. We performed short-term feeding experiments to examine ingestion, accumulation, biotransformation, histopathology, and paralysis in the juvenile Pacific calico scallop *Argopecten ventricosus* that consume this dinoflagellate. Depletion of algal cells was measured in closed systems. Histopathological preparations were microscopically analyzed. Paralysis was observed and the time of recovery recorded. Accumulation and possible biotransformation of toxins were measured by HPLC analysis. Feeding activity in treated scallops showed that scallops produced pseudofeces, ingestion rates decreased at 8 h; approximately 60% of the scallops were paralyzed and melanin production and hemocyte aggregation were observed in several tissues at 15 h. HPLC analysis showed that the only toxins present in the dinoflagellates and scallops were the *N*-sulfo-carbamoyl toxins (C1, C2); after hydrolysis, the carbamate toxins (epimers GTX2/3) were present. C1 and C2 toxins were most common in the mantle, followed by the digestive gland and stomach-complex, adductor muscle, kidney and rectum group, and finally, gills. Toxin profiles in scallop tissue were similar to the dinoflagellate; biotransformations were not present in the scallops in this short-term feeding experiment.

## 1. Introduction

The dinoflagellate *Gymnodinium catenatum* is responsible for red tides in many localities along the Pacific coast of Mexico [1,2,3,4,5,6,7,8,9]. This planktonic dinoflagellate produces paralyzing shellfish poisons (PSP) that include more than 20 neurotoxic, hydrophilic, tetrahydropurine derivatives [10,11,12] that make up four subgroups: (1) carbamates (STX, neoSTX, and gonyautoxins (GTX1–GTX4)); (2) *N*-sulfo-carbamoyl (GTX5, GTX6, C1–C4); (3) decarbamoyl (dcSTX, dcneoSTX, and dcGTX1–dcGTX4); and (4) deoxydecarbamoyl (doSTX, doneoSTX, and doGTX1) [13]. The action mechanism of PSP in vertebrates is the competitive block of sodium channels in excitable cells that inhibit influx of sodium ions, which in turn produces muscle paralysis or death in the most severe situation [14,15].

Bivalve mollusks are filter feeders that consume toxic dinoflagellates, concentrating and accumulating toxins in their tissues. The shellfish become potentially toxic to other animals [11,16]. Incidents of poisoning and death in humans in Mexico (1970–2004) are principally associated with consuming bivalves that ingested *Pyrodinium bahamense* and *G. catenatum* [17]. Worldwide, dinoflagellate poisoning is a constant threat to public health and has negative impacts on marine ecology, causing serious economic losses to aquaculture enterprises, fisheries, and tourist industries [18,19,20]. Many studies over the past several decades have shown that producers of PSP, such as *Alexandrium* spp. harm bivalves in a number of ways, such as reducing filtering, impairing valve activity, mantle retraction, mucus production, increased melanization of tissues, aggregating of hemocyte, decreased growth [18,21,22,23,24,25,26,27,28], and cause paralysis, as seen in vertebrates [29,30]; these physiological responses vary according to the type of toxic algae and the species of bivalve [31,32,33,34].

PSPs undergo chemical and enzymatic transformations that change one molecular form to another, becoming less or more toxic; this takes place in the dinoflagellate cell and the animals that consume the toxins. Although accumulation of PSP in bivalves is closely related to the mass of toxic dinoflagellates, bivalves frequently contain a higher proportion of carbamate toxins (or a lower proportion of *N*-sulfo-carbamoyl toxins) compared to the concentrations in the causative dinoflagellates [35,36]. The transformations are mediated principally by reductive cleavage (desulfation and dehydroxylation) and hydrolysis (decarbamoylation and desulfation). Absolute toxicity of PSP frequently varies, with carbamates as the most toxic [37,38,39,40,41]. Toxin levels in bivalves are a function of cell density, duration of exposure, toxicity and relative abundance of the phytoplankton species [11]. Differences in the dynamics of uptake of toxins and detoxification mechanisms, localization in organs, and physiological breakdown or transformation determine the persistence of toxins in tissues of bivalves [39,40,41].

The Pacific calico scallop *Argopecten ventricosus* is a suspension-feeding bivalve mollusk inhabiting the coast of the Baja California Peninsula of Mexico to the southern coast of Peru. In the Baja California Peninsula, the local commercial fishery for this scallop is of interest to aquaculturists. Ten PSP toxin analogs of several strains of *G. catenatum* in the Gulf of California and their presence in this scallop from Bahía Concepción (26.75° N, 111.8° W) have been described; the profile of toxins was similar in the scallops and the dinoflagellate [8,9]. In this study, we performed feeding trials under laboratory conditions to determine ingestion, PSP kinetics, paralysis, and histopathology in juvenile *A. ventricosus* that consumed *G. catenatum*.

## 2. Results and Discussion

### 2.1. Feeding Responses

**Figure 1:**
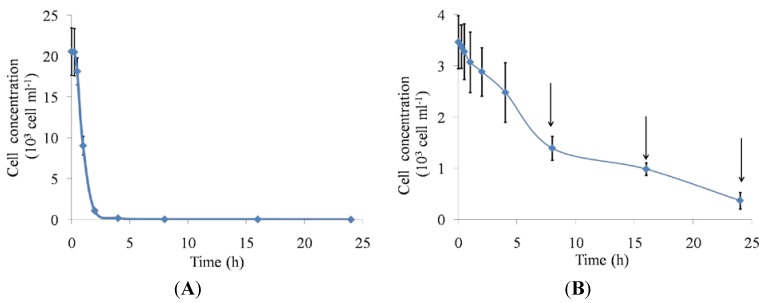
Depletion by juvenile Pacific calico scallops *Argopecten ventricosus* (shell height = 4.16 cm ± 1.1 cm) over 24 h of (**A**) *Isochrysis galbana* control cells and (**B**) toxic *Gymnodinium catenatum* cells. *G. catenatum* cells were given once at a concentration of 3500 cells mL^−1^ and *I. galbana* were given once at a concentration of 25 × 10^3^ cells mL^−1^ in a final volume of 1.5 L. Data are mean ± SD (control *n* = 48; treated *n* = 48). Arrows indicate production of pseudofeces.

The Pacific calico scallop *A. ventricosus*, exposed to toxic dinoflagellate cells, showed immediate behavioral responses after consuming *G. catenatum*, including partial shell valve closure, production of pseudofeces, and paralysis of the adductor muscle. Scallops fed toxic dinoflagellates have low feeding activity compared to scallops fed non-toxic microalgae (Figure 1). At 2 h, scallops in the control group had completely consumed their food, with peak ingestion at 30 min after the start of the experiment (Figure 1A). Scallops fed toxic dinoflagellates did not consume all of these cells after 24 h (Figure 1B). During the 0–2 h time frame, treated scallops filtered moderate quantities of the toxic dinoflagellate with partial shell valve closure; at 4 h, the scallops appeared to filtrate normally. There was a peak at 4 h associated with the opening of the shell valves, which suggested that normal functioning, with increasing filtration, was re-established. However, at 8 h, the scallops produced pseudofeces and ingestion rates decreased. Scallops can detect chemical and physical differences between particles by pre-ingestive and post-ingestive sorting [42,43]. Pseudofeces production has been shown to be a particularly important pre-ingestive mechanism because it not only prevents the animal’s ingestive capacity from being exceeded, but also facilitates the process of particle selection, whereby less nutritious particles may be rejected and the quality of the ingested material improved proportionately [44]. 

PSP is not the only toxin produced by these toxin producers that affect bivalve behavior. Recently, other toxins have been identify in *Gymnodinium* spp., such as the benzoate saxitoxin analogs [45,46,47,48,49], and gymnodimines [50,51,52,53] that could be present in our *G. catenatum* strain but were not detected with our method. Other PSP producers, such as *Alexandrium* spp., are also known to produce other toxic compounds, such as ichthyotoxins [54] and allelochemicals [55,56], and it has been reported that *A. minutum* showed potent toxic effects on brine shrimp *Artemia salina* [57] and a harpacticoid copepod *Euterpina acutifrons* [58], independent of PSP effects.

On the other hand, some scallops will preferentially retain larger particles longer than smaller ones, and lighter particles longer than denser ones [59]. The strain of *G. catenatum* used in our study can form chains of more than 60 organisms, reaching >1 mm in length. This could contribute to the avoidance feeding behavior of *A. ventricosus* because general patterns of size selectivity by bivalves show positive selection for particles above a lower limit of a few microns, which peaks for mid-sized particles, typically 20–30 µm, and particles above these sizes could be rejected [60].

### 2.2. Histopathological Findings

**Figure 2:**
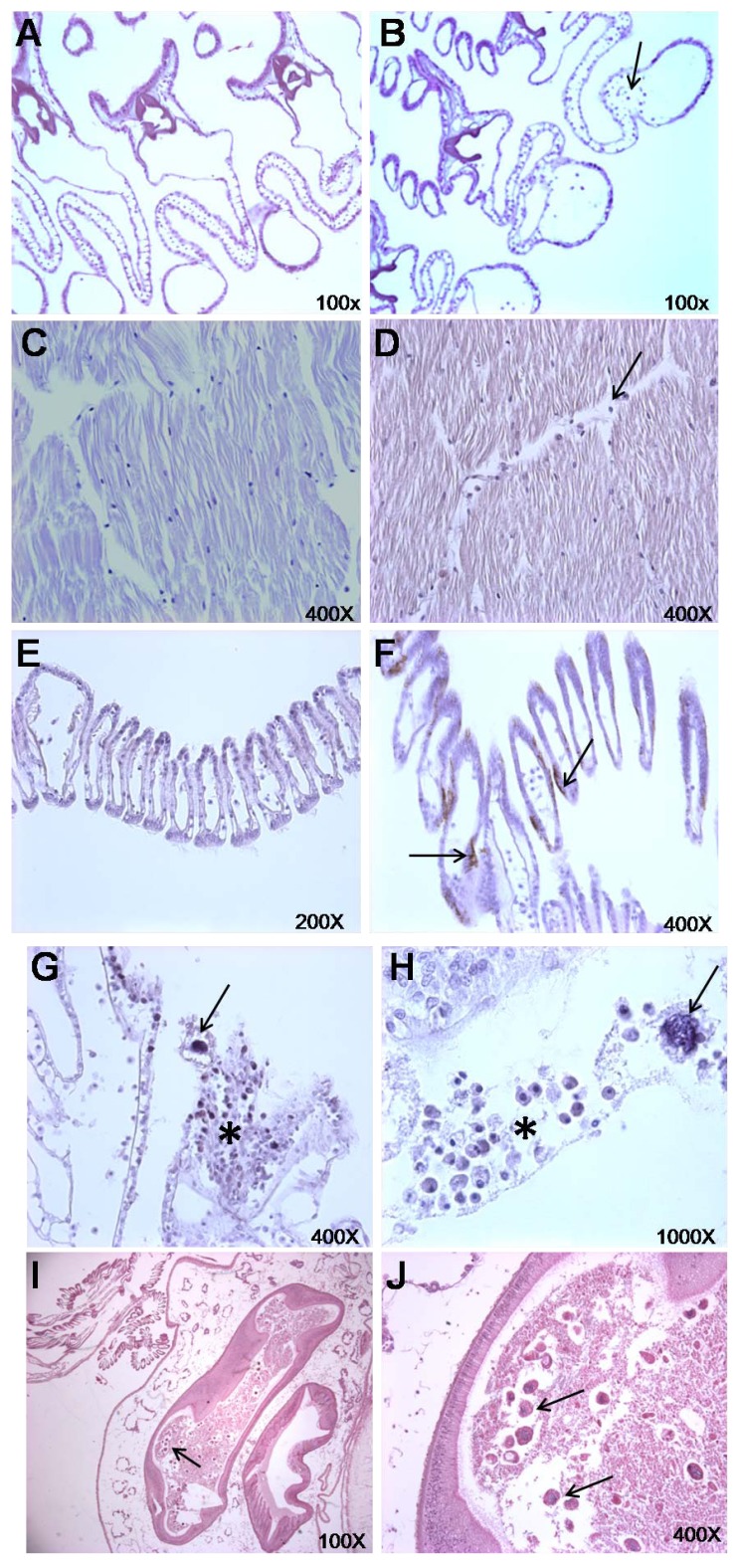
Histopathological microphotographs of *Argopecten ventricosus*, showing effects of exposure to the toxic dinoflagellate *Gymnodinium catenatum*. Exposed scallops (*n* = 12) (**B**, **D**, **F**, **G**, **H**, **I**, and **J**) and control scallops (*n* = 12) (**A**, **C**, and **E**). (**B**) Hemocyte aggregation in gill filaments; (**D**) Hemocyte aggregation in adductor muscle; (**F**) Gills with melanization; (**G**) Dinoflagellate in gills (arrow); (**H**) Dinoflagellate in mantle (arrow). Note the hemocyte aggregation (arrow) near the dinoflagellate (asterisk) and mucus production; (**I** and **J**) Dinoflagellates in the intestine and in the gonad (arrows).

Representative microphotographs of scallop tissues exposed to *G. catenatum* are displayed in Figure 2. Gills, adductor muscle, and mantle have more circulating hemocytes in treated scallops compared to the control scallops (Figure 2A–D,G–H). Some dinoflagellates were found in the gills and mantle; occasionally, accumulations of hemocytes aggregated near these dinoflagellates, surrounded with mucus (Figure 2G–H). We also observed epithelial melanization in mantle and gill tissue of scallops fed toxic dinoflagellates (Figure 2E). To test for statistical differences in histological samples, we performed chi-square analysis (χ^2^ test) to identify the relationship between the adverse effects in tissues of scallops by response to the toxic dinoflagellate *G. catenatum*, expressed in terms of categorical variables indicating the presence or absence of epithelial melanization, mucus production, and hemocyte aggregation (Table 1). Results show a strong association of these histological features in tissues of scallops exposed to *G. catenatum* cells as food. Epithelial melanization of gills and mantle showed a high positive response to *G. catenatum* (72.2% and 86.1%, respectively; *P* < 0.0001). Mucus production and accumulation of hemocytes in gills showed low percentage response to *G. catenatum* (44.4% and 30.6%, respectively), however these results were also significantly different compared with controls (*P* < 0.001). In the control group, histological observations of epithelial melanization in mantle and gills were almost negative, with a small percentage of scallops having a positive response (16.7% and 22.2%, respectively). 

**Table 1:** Results of contingency table analysis (χ^2^ test) relating epithelial melanization, mucus production, and hemocyte aggregation in a subset of histological sections from *Argopecten ventricosus* tissues (gills, mantle, and muscle) exposed to *Gymnodinium catenatum*, compared with scallops that were not exposed to toxic dinoflagellates.

	Variable	Negative		Positive		χ^2^	*P*
		*N*	%	*n*	%		
Gills							
	Epithelial melanization					22.5	<0.0001
	Control	30	83.4	6	16.6		
	Treated	10	27.8	26	72.2		
	Mucus production					14.5	0.0001
	Control	34	94.4	2	5.6		
	Treated	20	55.6	16	44.4		
	Hemocyte accumulation					10.0	0.0016
	Control	35	97.2	1	2.8		
	Treated	25	69.4	11	30.6		
Mantle							
	Epithelial melanization					29.6	<0.0001
	Control	28	77.8	8	22.2		
	Treated	5	13.9	31	86.1		
	Mucus production					22.8	<0.0001
	Control	31	86.1	5	13.9		
	Treated	11	30.6	25	69.4		
	Hemocyte accumulation					22.4	<0.0001
	Control	35	97.2	1	2.8		
	Treated	17	47.2	19	52.8		
Muscle							
	Hemocyte accumulation						
	Control	33	91.7	3	8.3	22.1	<0.0001
	Treated	14	38.9	22	61.1		

*n* = number of sections observed from 12 treated scallops and 12 controls.

In mollusks and other invertebrates, host defense mechanisms and homeostasis are modulated by circulating hemocytes that ingest and recognize invading microorganisms and engage in phagocytosis, inflammation, and wound repair [61,62]. Accumulation of hemocytes in tissues to attack toxic marine microorganisms has been observed numerous times in bivalves [26,29,30,63]. Mucus is important during ingestion of particles; mucocytes are present in tissue surfaces involved in ingesting and transporting particles and as a first line of defense against pathogens [64,65,66]. Synthesis of melanin is part of the defense against pathogens; the pigment eliminates pathogens, as well as acting in a chain of defensive reactions involving phenoloxidase [67]. Dinoflagellate cells were also observed in intestinal tissue in all treated scallops (Figure 2I–J). Toxic cells could be ingested, but not digested, and pass through the digestive system intact in feces [68,69]. In the juvenile giant lions-paw scallop *Nodipecten subnodosus*, which naturally occur with Pacific calico scallops, *G. catenatum* are consumed and lions-paw scallops respond, at high concentrations of these cells, by producing pseudofeces, partially closing their shell valve, reducing feeding rates, increasing melanization, and aggregating hemocytes [26]. Bivalve responses to marine toxic algae are species-specific and depend upon a variety of factors, including the algal species encountered, algal toxicity, algal concentration, cell size and selectivity, history of exposure, season, and differences in digestive function [18,21,22,28,39,40,41,63,70,71].

### 2.3. Paralysis of Scallops

**Figure 3:**
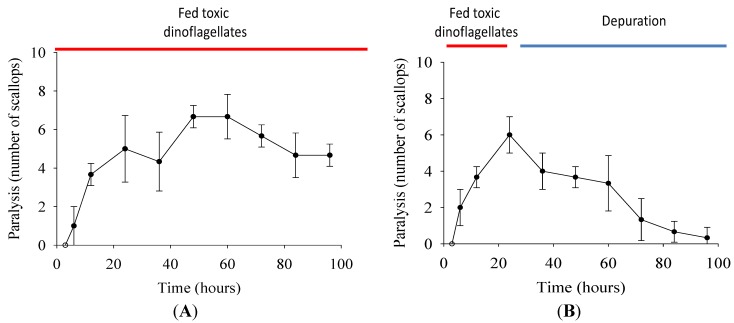
Juvenile scallops (shell height = 4.03 cm ± 0.4 cm): (**A**) fed 4000 cells mL^−1^ *Gymnodinium catenatum* for 96 h, and (**B**) fed 4000 cells mL^−1^ *G. catenatum* cells for 24 h and afterward fed 10 × 10^3^ cell mL^−1^ non-toxic microalgae *Isochrysis galbana*. Paralysis and recovery time were recorded for each individual. In both treatments, data were obtained at 3, 6, 12, 24, 36 48, 60, 72, 84, and 96 h. Control scallops were fed 25 × 10^3^ cell mL^−1^ non-toxic microalgae *I. galbana*, also for 96 h, and no paralysis was observed. Data represent mean ± SD (control *n* = 20; treated *n* = 30).

Scallops were paralyzed at 15 h when fed 4000 cells mL^−1^ of *G. catenatum* and recovered hours later. We recorded onset time of paralysis and recovery under two different feeding regimens (Figure 3). When scallops were fed *G. catenatum* for 96 h, ~60% of the scallops were paralyzed, only some recovered (Figure 3A) and 10% died. When scallops were fed *G. catenatum* for 24 h, followed by non-toxic microalgae, most scallops recovered by 96 h; only a few remained paralyzed (Figure 3B), and none died. The scallops fed non-toxic microalgae (control group) were not paralyzed and are not shown in results. Paralysis of the adductor muscle is well known in oysters [29]. Injecting PSP intramuscularly in *Nodipecten subnodosus* induces paralysis; these scallops gradually recover in a dose-time recovery pattern [30], similar to our observations of shell closure and normal feeding responses, as indicators of recovery.

**Figure 4:**
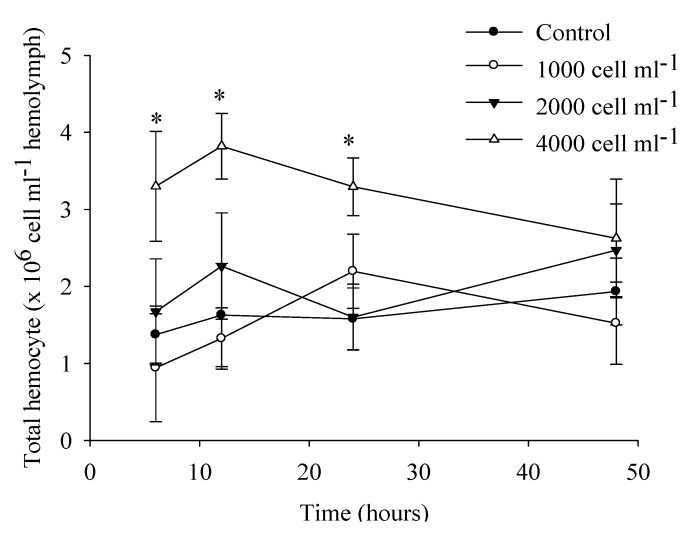
Hemocyte counts over 48 h in juvenile Pacific calico scallops *Argopecten ventricosus* (shell height = 4.98 cm ± 0.7 cm) fed different concentrations of *Gymnodinium catenatum* under controlled conditions. Hemolymph was withdrawn and counted at 6, 12, 24, and 48 h. At the highest concentration of toxic alga cells, some scallops were paralyzed. Paralyzed and non-paralyzed scallops were tested. Data represent mean ± SD (control *n* = 10; treated *n* = 10). Asterisk indicates statistically significant differences between the control and treated groups (*P* < 0. 05).

Hemocytes increased when scallops were fed high concentrations of toxic dinoflagellates (4000 cells mL^−1^; Figure 4), which was markedly different from the control group. About 25% of treated scallops became paralyzed; below this concentration, scallops were not significantly different from the control group. Although we did not use a long-period feeding treatment, toxic dinoflagellates could produce immunosuppression in the scallops, as was the case in *N. subnodosus* when toxins were injected into the adductor muscle (100 mouse units; MU), permanent paralysis occurs and hemocytes in hemolymph decreased [30]. However when these scallops were fed toxic dinoflagellates, they were not paralyzed [25,26]. When several species of bivalves received PSP by intramuscular injection, the minimum lethal dose was remarkably high, 300 MU 20 g^−1^, compared to the sensitivity of fish at 0.5–33 MU 20 g^−1^ and crustaceans at 0.5–10 MU 20 g^−1^ [72,73].

Other effects produced by PSP in bivalves are the absence of response to mechanical stimulation of the gills and adductor muscle in gaping *Crassostrea virginica* [29], incapacity to burrow, associated with muscle paralysis, in the soft-shell clam *Mya arenaria* [74], paralysis in adductor muscle after spawning events in mussels [69] and important morphological alterations of the adductor muscle in *C. gigas* [75]. Although scallops in our work become paralyzed, scallops do not die and this resistance in some bivalve species is related to differences in toxin accumulation, where resistant species, such as *Mytilus edulis*, accumulate toxins in tissues at greater rates than sensitive bivalves [11,76]. Reduction in nerve sensitivity in clams is the result of natural mutations of amino acid residues that decreases the affinity of the saxitoxin-binding site in the sodium channel pore [77], which may explain why there are PSP-resistant and non-resistant bivalve species. Highly-sensitive species, such as the hard clam *Mercenaria mercenaria* and the bay scallop *A. irradians*, exhibit physiological and behavioral mechanisms to avoid toxicity by reducing filtration rate and rapid shell clapping to clear the gills of toxic cells [21].

### 2.4. Analysis of Toxins

Composition of PSP derivatives in *G. catenatum* cells, before chemical hydrolysis, was limited to *N*-sulfo-carbamoyl toxins (C1 and C2). The toxin profiles of dinoflagellates isolated from various areas have been elucidated by many research groups. These studies indicate that the toxin profile is often specific to a species of dinoflagellate. According to Oshima *et al.* [35] *G. catenatum* shows a characteristic toxin profile which consists of *N*-sulfo-carbamoyl toxins such as B and C toxins as major components.

The toxin content of different tissues we found in *A. ventricosus* also showed that the principal PSP native components were C1 and C2 toxins. It is common that bivalve tissues transform *N*-sulfo-carbamoyl from toxic dinoflagellates to more toxic carbamates toxins. Various C-toxins undergo chemical and enzyme-mediated reductive cleavage (desulfuration and dehydroxylation) and hydrolysis (decarbamoylation and desulfuration) *in vivo* [78,79,80,81]; biotransformations sometimes cause increased toxicity, compared to intact toxins transmitted by dinoflagellates [80]. Only C1 and C2 toxins were present in *G. catenatum* and *A. ventricosus* tissues in our short-term feeding trial, which indicated a low capacity of transformation to carbamate toxins *in vivo*, similar to results observed in samples from the field by Band-Schmidt *et al*. [8] with the same species. Band-Schmidt *et al.* [8] principally found ten toxins in *G. catenatum* cells and samples of *A. ventricosus*, but only dcSTX, dcGTX2, dcGTX3, C1, and C2 were always present in all strains of *G. catenatum* at both growth stages and in the profiles in *A. ventricosus*. Although Band-Schmidt *et al.* [8] used the same protocol for toxin extraction, and the toxic dinoflagellates come from similar areas, we did not detect decarbamoyl toxins. The total toxin content in *G. catenatum* in our study was similar to described PSP profiles of 16 isolated and cultured strains of *G. catenatum* from the Gulf of California, where the average toxicity of *G. catenatum* strains was 26 ± 6 after 17 days of growth and 28 ± 18 pg STXeq cell^−1^ after 22 days of growth. Differences in toxin profiles in bivalves and in dinoflagellates might be explained by cell culture conditions, specific uptake, elimination, and enzymatic and chemical transformations of accumulation in tissues, and in the methods used [82,83,84,85,86,87,88,89].

To analyze whether *N*-sulfo-carbamoyl toxins could convert to carbamate toxins, we examined the possibility of transformation of these PSP analogs performing a chemical hydrolysis with 1 M HCl; it also serves to make comparisons of toxin concentration with the analytical data obtained with other works that use direct hydrolysis to report their results. Different extraction conditions may lead to differences in toxin profiles and to different results when these analytical data are expressed as STX equivalents. After chemically-induced hydrolysis, we detected carbamate toxins and epimers GTX2/3 in *G. catenatum* cells and *A. ventricosus* tissues. PSP are prone to various conversions, depending on the pH; when heated at low pH, toxins with the *N*-sulfo-carbamoyl moiety as a side chain are partially converted to the corresponding carbamate toxins through hydrolysis [13]. These conversions take place when PSP are boiled with strong acid and result in a change of PSP analogues with a low toxicity, such as C1 and C2, into ones with a higher toxicity, such as GTX2 and GTX3. Conversion does not take place in weak acids, such as acetic acid solutions, ~pH 3–4. Because the Cs toxins we analyzed could be converted to their respective carbamate analogs, in long-term trials, *in vivo* biotransformations may occur in *A. ventricosus*.

Table 2 shows accumulation of PSP in different tissues of *A. ventricosus.* No toxins were detected in tissues of the control group, and we did not measure other toxins that could be present in *G. catenatum*. In *A. ventricosus* before hydrolysis, the mantle had the highest concentration of toxins, followed by the digestive gland-stomach complex, gills, and finally by the muscle, kidney, and rectum group. Generally, the tissues that accumulated the most PSP are in contact with food particles when they are ingested and during digestion. Commonly, the digestive gland is the tissue that accumulates most of the toxin body burden, similar to other scallops, such as *Placopecten magellanicus*, *Patinopecten yessoensis*, and *Nodipecten subnodosus* [25,83,84,85]. Since the digestive gland carries out digestive processes and transformation of toxins, the digestive gland-stomach complex is probably the most important tissue in toxin metabolic processes [29,35]. Thus, accumulation of PSP depends on the capacity for biotransformation and selectivity among tissues and variations among individuals [10,89,90].

**Table 2:** Accumulation of PSP in different tissues of *Argopecten ventricosus* (pg STX eq g^−1^ wet weight) and in *Gymnodinium catenatum* cells (pg STX eq cell^−1^).

Extract	C1	C2
*Gymnodinium catenatum*	45 ± 6	15 ± 6
Tissue controls	ND	ND
Digestive gland-stomach	38 ± 3	189 ± 13
Mantle	41 ± 8	202 ± 37
Gills	10 ± 1	51 ± 6
Muscle, kidney, rectum	11 ± 2	56 ± 8

Data from tissues represent mean ± SD (control *n* = 12; treated *n* = 12). ND = Not detected.

Band-Schmidt *et al.* [91] reviewed PSP events from *G. catenatum* in the Pacific areas of Mexico. Low toxicities (<1000, >50 μg STXeq 100 g^−1^) were detected in *A. ventricosus* [92,93], compared with high toxicities (>1000 μg STXeq 100 g^−1^) found in oysters and mussels [94,95,96,97]. Some authors claim that accumulation profiles of PSP toxins in bivalves differ significantly among bivalve species [97,98,99]. For example, during toxic algal blooms, the scallops *P. yessoensis* and *Chlamys nipponensis* become much more toxic than mussels under the same environmental conditions, and their depuration requires several months. Mussels and oysters usually accumulate high levels of toxicity rapidly and also decline rapidly once the causative dinoflagellates disappear. Thus, the season of sampling, concentrations of algal cells, and physicochemical parameters are important factors in determining dinoflagellate and bivalve toxicity [91]. 

## 3. Experimental Section

### 3.1. Algal Culture and Source of Scallops

*Gymnodinium catenatum* strain GCQM-2 was obtained from the Marine Dinoflagellate Collection (CODIMAR) at CIBNOR in La Paz, Mexico [100]. The cells were cultured in GSe medium [101] prepared with filtered (0.7 μm) seawater in 20 L glass flasks on a 16 h light: 8 h dark photocycle at 21 °C under 70 W fluorescent lamps and anaerobic conditions.

Cultivated juvenile scallops (shell height = 4.2 ± 1.1 cm) were collected from farms at Rancho Bueno, Baja California Sur, Mexico (24°32′ N, 111°42′ W) and transported to CIBNOR. Groups of 12 were placed in a 40 L plastic tank containing 1μm filtered seawater. The water was maintained at 22 °C, with salinity at 35 practical salinity units (psu), and constantly aerated with air stones. Filtered seawater was completely replaced every two days. During acclimation (14–21 days), the scallops were fed a mix of microalgae (*Chaetoceros calcitrans*, *Chaetoceros gracilis*, and *I. galbana*; 1:1:1 ratio, 200 × 10^3^ cells mL^−1^ of each), obtained from the Microalgae Laboratory at CIBNOR. *C. calcitrans* and *C. gracilis* were cultured in 20 L plastic bags in F/2 growth medium at 22 °C with constant illumination at salinity of 32 psu. *I. galbana* strain ISG-1 was grown in MA-F/2 medium at 22 °C at 32 psu in 20 L plastic bags under constant illumination. *I. galbana* was also used as the non-toxic diet for the control group. *G. catenatum* cultures were harvested during the late exponential growth phase; the three microalgae were harvested during the stationary growth phase. To estimate concentrations at harvest, cells were counted in Sedgewick-Rafter counting chambers under an optical microscope (400×) for the dinoflagellates and an electronic particle counter (Coulter Multi-sizer) for the other microalgae.

### 3.2. Feeding Experiments

Three feeding experiments were performed: (1) A 24 h trial at one concentration of *G. catenatum* to study depletion of *G. catenatum*, accumulation and biotransformation of PSP, and observe histological changes; (2) A 48 h feeding experiment with different concentrations of *G. catenatum* to analyze number of hemocytes in *A. ventricosus*; and (3) A 48 h feeding experiment at a high concentration of *G. catenatum* to measure paralysis and recovery time of scallops. For filtration of *G. catenatum* and accumulation and biotransformation of PSP, 36 scallops were used; for histopathology, 12 scallops; for total hemocytes count, 80 scallops; and for paralysis studies, 100 scallops. Before the trials, specimens were placed in 1 μm filtered seawater without food for 24 h to clear their digestive tract.

#### 3.2.1. Experiment 1

To assay accumulation and biotransformation of PSP, 48 juvenile scallops (shell height = 4.2 ± 1.1 cm) were used and 24 scallops for histopathology study. Groups of six scallops were placed in six 1.5 L plastic containers. Each group was fed 3500 cells mL^−1^ *G. catenatum* in a volume of 1.5 L filtered seawater at the beginning of the trial under closed, controlled conditions (22 °C, 35 psu), and responses were observed for 24 h. Another six 1.5 L plastic containers, each with six scallops, were used as the control group and fed *I. galbana* (25 × 10^3^ cells mL^−1^). To reduce cell growth during the experiment, algae were maintained in the dark and kept in suspension with a bubbling air stone.

#### 3.2.2. Experiment 2

Groups of five juvenile scallops were placed in 1.5 L plastic containers, four containers for each concentration of *G. catenatum*. Four additional containers were used for the control group, which were fed *I. galbana* (25 × 10^3^ cells mL^−1^) at the beginning of the experiment. Scallops (shell height = 5 ± 0.7 cm) were fed one of three concentrations (1000, 2000, or 4000 cells mL^−1^) of *G. catenatum* in a volume of 1.5 L filtered seawater at the beginning of the trial; the water was completely changed at 24 h; the same concentrations of *G. catenatum* was added for the next 24 h of the trial (22 °C, 35 psu). Five scallops from one control container and 5 scallops from one treated container were used at 6, 12, 24, and 48 h for hemolymph extraction. Hemolymph was withdrawn and hemocytes counted (see below). Hemocyte counts were recorded for each individual and no scallop was withdrawn more than once. At the highest concentration of toxic alga cells, some scallops were paralyzed; paralyzed and non-paralyzed scallops were tested.

#### 3.2.3. Experiment 3

Juvenile scallops (shell height = 4 ± 0.4 cm) were fed 4000 cells mL^−1^ *G. catenatum* in a volume of 1.5 L filtered seawater under closed, controlled conditions (22 °C, 35 psu). The seawater was completely changed every 24 h for three days. The same concentration of *G. catenatum* was added each day to the replacement seawater. Ten scallops were placed in 1.5 L plastic containers. Three containers were used in this trial; two other containers with 10 scallops each were fed the non-toxic microalga *I. galbana* (25 × 10^3^ cells mL^−1^). The onset of paralysis and recovery time of treated scallops was observed and recorded for 96 h. A similar trial was carried out in parallel, but *G. catenatum* was added only at the beginning of the experiment, and at 24 h; the scallops were fed the non-toxic microalgae *I. galbana* at a lower concentration (10 × 10^3^ cell mL^−1^) to record onset of paralysis and recovery time over the following 72 h. Previously, we observed that, when scallops are paralyzed, they continue to feed at a reduced level of activity. This is the basis for feeding them a low concentration of non-toxic microalgae. In both cases, data were recorded at 3, 6, 12, 24, 36, 48, 60, 72, 84, and 96 h.

### 3.3. Depletion of Algal Cells

Depletion of algal cells in Experiment 1 was measured in the closed systems. Samples of the suspension (1 mL) from each container, fixed in Lugol’s solution (1:1, v:v), were counted under an optical microscope or in the electronic particle counter at the beginning of exposure (0 h) and at 0.25, 0.5, 1, 2, 4, 8, 16, and 24 h after exposure. Scallops of eight containers (control *n* = 24; treated *n* = 24) were sacrificed at 24 h to determine total wet tissue weight, shell size and toxin content.

### 3.4. Histopathological Examination

Scallops from another four containers from Experiment 1 were processed for histopathology examination (control *n* = 12; treated *n* = 12). Whole animals were removed from their shells and preserved in Davidson’s solution. Samples of tissues were embedded in paraffin, sectioned to 5 μm, and stained in hematoxylin-eosin. Permanent slides were examined under an optical microscope and recorded with digital micrographs.

#### Contingency Tables

To understand the relationship between histological type and response to PSP, we made contingency tables with the following characteristics: (1) Histological type: epithelial melanization, mucus production, hemocyte accumulation; (2) Response: positive and negative; and (3) Frequency: number of organisms in a given histological type and response category. With these data, we used the chi-square test (χ^2^ test) with statistical software (JMP 7, SAS Institute, Cary, NC, USA). Data come from three random histological sections of each scallop, which included gills, mantle, and adductor muscle cut longitudinally from the middle of the adductor muscle with a difference of approximately 50–80 μm between sections. For each histological section, the percentage of the responses was tabulated as the coverage area of the effect (presence of brown pigment melanin and occurrence of mucus) or as accumulation of hemocytes (frequency of number of aggregations). The area occupied by melanin and mucus in epithelia was determined with an image analyzer (Image Pro Plus v4, Media Cybernetics, Bethesda, MD, USA) at 40× (7.9 mm^2^) from three different slices from each specimen, similar to the method described by Rodríguez-Jaramillo *et al.* [102]. Ten images were taken randomly in each tissue from each section and image analyses were based on the intensity of the tissue-specific color (melanin) and the area that was manually selected (mucus), which were automatically calculated in pixels and expressed in μm^2^. The reported response coverage area for each effect was calculated as: RCA = (occupation area affected/total area) × 100. A positive response was indicated when the RCA covered >50% (from a mean of the ten images); a negative response was indicated when the RCA was <50%. Accumulation of hemocytes was positive when 5 or more aggregations were observed.

### 3.5. Paralysis Studies

#### 3.5.1. Determination of Paralysis

In feeding experiments, some scallops were paralyzed and recovered hours later. In Experiment 3, kinetics of paralysis was assessed, as described by Hégaret *et al.* [29] after appearance of a gaping shell. Gaping scallops were mechanically stimulated on the gills and adductor muscle with a micropipette; if the scallops did not react with movement, they were considered paralyzed. Viability of paralyzed scallops was determined by examining the clarity of the water and production of feces after feeding low concentrations of non-toxic microalgae. Recovery of paralyzed scallops was determined by observation of reduced shell gaping and recovery of muscle function.

#### 3.5.2. Hemocytes Count at Different Toxic Cells Concentrations

To extract hemolymph in Experiment 2, each scallop’s valves were pried open and kept separated with a knife blade; the hemolymph was withdrawn from the adductor muscle with a 26 gauge needle attached to a 1 mL syringe. Approximately 0.5–1.0 mL hemolymph per scallop was drawn and immediately put on ice to avoid clumping. Total hemocyte count was recorded with an electronic particle counter (Coulter Multi-sizer). This experiment was done twice and data from both trials were analyzed together by the Student’s *t*-test with SPSS 16 software (eight containers per concentration at each time, see section 3.2.2). Significance was set at *P* < 0.05.

### 3.6. Analysis of Toxins

#### 3.6.1. Algal and Scallop Toxins

Two liters of *G. catenatum* culture were harvested for analysis by extracting the biomass by centrifugation at 500× *g*. The precipitate was suspended in 50 mL 0.05 M acetic acid and then homogenized with glass beads (5 mm dia.). Three successive washes by stirring were done (30 s vortex: 30 s ice). The supernatants were pooled. Examination under an optical microscope of the remaining cell debris after homogenization revealed that the cells had been completely disrupted. Toxins were stored at −80 °C.

To study the accumulation and biotransformation of toxins, the scallops from three of the containers from Experiment 1 were used at the end of the one-day exposure. All scallops in each container were weighed, pooled, and stored at −80 °C. About 5–10 g of tissue was emulsified in 0.05 M acetic acid in proportions of 1:2 (tissue weight:acid volume) in a homogenizer (Polytron, Kinematica, Bohemia, NY, USA). The suspension was centrifuged for 10 min at 5000× *g*, and the supernatant was filtered with a single-use syringe-filter (0.2 µm). The extracts (cells and tissues) containing PSP were combined with 150 mL 0.03 N acetic acid and 37 µL 1 M HCl and mixed in a vortex for 30 s and then hydrolyzed by heating for 15 min at 90 °C. The mixture was neutralized with 75 µL (1 M sodium acetate) and stirred for 30 s. Samples were cooled to room temperature.

#### 3.6.2. HPLC Analysis

PSP were measured under the conditions described previously, using post-column derivatization HPLC with the fluorescent online detection method [78,79]. Briefly, a 20 μL pretreated sample was injected into a reverse phase column C18 (4.6 mm ID, 5 μm particle size, 25 cm long, and 200 Å pore size) with a mobile phase composed of a solution of 11 mM octanesulfonic acid and 40 mM phosphoric acid with pH adjusted to 6.9 with ammonia phosphate in 15% tetrahydrofurane. Column eluted fractions were mixed with 10 mM periodic acid in 550 mM buffered ammonia at pH 9.0 at 0.3 mL min^−1^, heated to 50 °C by passing through a coil of Teflon tubing (0.2 mm ID, 10 cm long), and then mixed with 1 M nitric acid at a flow rate of 0.4 mL min^−1^ just before entering the monitor. The fluoromonitor was set to excite at 330 nm and emit at 395 nm. Detection limits for the method are 247, 17, 77, 16, and 7 pg mL^−1^ for GTX1, 4 pg mL^−1^ for GTX2, and 3 pg mL^−1^ NeoSTX, dcSTX, and STX. PSP were identified by comparing chromatograms obtained from samples with standard solutions of STX, NeoSTX, GTX1, GTX2, GTX3, GTX4, and dcSTX (National Research Council Canada, Institute for Marine Biosciences, Certified Reference Materials Program, Halifax, NS). Quantification of PSP content used the factor response (peak area/toxin concentration) obtained with the injection of known quantities of toxin standards. Toxicity in tissues is expressed as picograms of saxitoxin equivalents g^−1^ scallop meat (pg STXeq g^−1^), that is, the relative toxicity of each derivative with respect to potential poison, namely saxitoxin (STX). Toxicity in cell is expressed in pg STXeq cell^−1^.

## 4. Conclusions

Similar physiological reactions occur in *Argopecten ventricosus*, as in other scallops, when ingesting the toxic dinoflagellate *Gymnodinium catenatum*, including reduced filtering, melanization of epithelial cells, and aggregation of hemocytes; however, paralysis of the adductor muscle generally had not been regarded as important in other scallops feeding on dinoflagellates that produce PSP. After initial consumption of toxic dinoflagellates, high concentrations of *N*-sulfo-carbamoyl toxins (epimers C1 and C2) occurred in the mantle and digestive gland-stomach complex and, to a lesser extent, in the gills and the adductor muscle-kidney-rectum group. We only found epimers of C1 and C2 in the short-term trial, suggesting that biotransformation in different tissues of this scallop is not obvious, as in other bivalves.
